# Crosstalk Between Dysfunctional Mitochondria and Inflammation in Glaucomatous Neurodegeneration

**DOI:** 10.3389/fphar.2021.699623

**Published:** 2021-07-21

**Authors:** Assraa Hassan Jassim, Denise M. Inman, Claire H. Mitchell

**Affiliations:** ^1^ Department of Basic and Translational Science, University of Pennsylvania, Philadelphia, PA, United States; ^2^ Department of Pharmaceutical Sciences, North Texas Eye Research Institute, University of North Texas Health Science Center, Fort Worth, TX, United States; ^3^ Department of Ophthalmology, University of Pennsylvania, Philadelphia, PA, United States; ^4^ Department of Physiology, University of Pennsylvania, Philadelphia, PA, United States

**Keywords:** retinal ganglion cells, mitophagy, microglia, glaucoma, NLRP3 infammasome, astrocyte, metabolic vulnerability

## Abstract

Mitochondrial dysfunction and excessive inflammatory responses are both sufficient to induce pathology in age-dependent neurodegenerations. However, emerging evidence indicates crosstalk between damaged mitochondrial and inflammatory signaling can exacerbate issues in chronic neurodegenerations. This review discusses evidence for the interaction between mitochondrial damage and inflammation, with a focus on glaucomatous neurodegeneration, and proposes that positive feedback resulting from this crosstalk drives pathology. Mitochondrial dysfunction exacerbates inflammatory signaling in multiple ways. Damaged mitochondrial DNA is a damage-associated molecular pattern, which activates the NLRP3 inflammasome; priming and activation of the NLRP3 inflammasome, and the resulting liberation of IL-1β and IL-18 via the gasdermin D pore, is a major pathway to enhance inflammatory responses. The rise in reactive oxygen species induced by mitochondrial damage also activates inflammatory pathways, while blockage of Complex enzymes is sufficient to increase inflammatory signaling. Impaired mitophagy contributes to inflammation as the inability to turnover mitochondria in a timely manner increases levels of ROS and damaged mtDNA, with the latter likely to stimulate the cGAS-STING pathway to increase interferon signaling. Mitochondrial associated ER membrane contacts and the mitochondria-associated adaptor molecule MAVS can activate NLRP3 inflammasome signaling. In addition to dysfunctional mitochondria increasing inflammation, the corollary also occurs, with inflammation reducing mitochondrial function and ATP production; the resulting downward spiral accelerates degeneration. Evidence from several preclinical models including the DBA/2J mouse, microbead injection and transient elevation of IOP, in addition to patient data, implicates both mitochondrial damage and inflammation in glaucomatous neurodegeneration. The pressure-dependent hypoxia and the resulting metabolic vulnerability is associated with mitochondrial damage and IL-1β release. Links between mitochondrial dysfunction and inflammation can occur in retinal ganglion cells, microglia cells and astrocytes. In summary, crosstalk between damaged mitochondria and increased inflammatory signaling enhances pathology in glaucomatous neurodegeneration, with implications for other complex age-dependent neurodegenerations like Alzheimer’s and Parkinson’s disease.

## Glaucomatous Neurodegeneration

Glaucoma is a neurodegenerative disease that can ultimately lead to irreversible blindness (Tham et al., 2014). This etiologically complex optic neuropathy is characterized by progressive structural and functional loss of retinal ganglion cells (RGCs). Pathology is found in RGC compartments; the soma in the inner retina, the axons which form the core component of the optic nerve head (ONH) and the optic nerve (ON) relaying visual information to the brain. The retina, ONH, ON, and brain regions respond differently in glaucoma, giving rise to compartmentalized degeneration (Tamm et al., 2017). Accordingly, RGCs can execute autonomous degeneration to eliminate different parts of themselves upon insult, including the dendrites and soma within the retina itself, the axons passing through the ONH and ON, and the synapses within the brain (Whitmore et al., 2005).

Many forms of glaucoma are associated with elevated intraocular pressure (IOP). While lowering IOP is currently the primary therapy available to slow down glaucoma pathology, it does not necessarily prevent blindness, and glaucomatous neurodegeneration extends beyond IOP elevation into complex cellular pathologies. The moderate elevations in IOP associated with most common forms of glaucoma, with IOP values 21–30 mmHg, are largely asymptomatic, resulting in a delayed glaucoma diagnosis, which in turn defers therapy initiation until after RGC death has begun. Normal tension glaucoma (NTG) can occur in individuals with IOP in the normal range of 15–20 mmHg; however, patients still benefit from lowering IOP suggesting a differential pressure sensitivity to IOP among individuals (Josef et al., 2002; Whitmore et al., 2005; Calkins, 2012). Regardless of IOP levels, glaucomatous neurodegeneration involves a complex interaction between multiple factions including age, genetics, mechanical strain, hypoxia, neurochemical signaling, autophagy, cellular energetics and immune signals. In this review, we will discuss the crosstalk between mitochondrial dysfunction and inflammation during glaucoma.

## Models of Glaucomatous Neurodegeneration

Molecular mechanisms of glaucoma differ from person to person and across animal models (Fernandes et al., 2015; De Moraes et al., 2017; Pang and Clark, 2020). All models require certain compromises, there is no “perfect” model of glaucoma and thus comparing results from multiple models provides a better understanding of glaucoma pathology. As rodent models facilitate large-scale studies and genetic manipulations, they offer convenience as model systems.

The DBA/2J (D2) mouse is a well-established model of inherited glaucoma (Libby et al., 2005). IOP elevation is secondary to excessive iris pigment dispersion, which consequently blocks the trabecular meshwork and drives aqueous humor accumulation, thereby causing IOP elevation. This iris disease is linked to recessive mutations in tyrosinase-related protein 1 (Tyrp1) and glycoprotein nonmetastatic melanoma B (Gpnmb). These mutations induce melanogenesis toxicity and a subsequent inflammatory response directed at the iris, which forms structural melanosome abnormalities seen in both humans and D2 mice. IOP elevation in D2 mice is spontaneous and progressive, starting at 6 months (14–18 mmHg) and leveling off by 11 months at a value of about 28 mmHg (Mahesh et al., 2007).

Ocular hypertension (OHT) can also be induced in animal models through a variety of surgical interventions to impede aqueous outflow to some extent and hence elevate IOP. Transient IOP elevations are produced by cannulating the anterior chamber of the eye, while more sustained elevations in IOP can be produced following hypertonic saline injection into the episcleral vein, microbead injection into the anterior chamber of the eye, translimbal laser photocoagulation, or cauterization of episcleral veins. The microbead model offers advantages in the mouse given its flexibility and consistency that allows relative ease of use (Sappington et al., 2010; Samsel et al., 2011; Yang et al., 2012), although the best choice is based on the specific experimental questions being addressed. IOP can also be elevated following steroid application (Whitlock et al., 2010; Overby and Clark, 2015); systemic administration of dexamethasone has been used to demonstrate the role of dopamine and serotonin in IOP regulation (Bucolo et al., 2012; Platania et al., 2013), and has even been used to raise IOP in cows (Gerometta et al., 2004). The combined use of multiple models to confirm a specific outcome is preferable given the inherent compromises with each.

## Mitochondrial Dysfunction in Glaucoma

Mitochondrial dysfunction has been strongly implicated in glaucomatous neurodegeneration in patients and multiple models of glaucoma (Kong et al., 2009; Munemasa et al., 2010; Lee et al., 2011; Kamel et al., 2017). Neurons are particularly sensitive to mitochondrial challenge as they require high levels of energy to maintain the electrochemical gradients necessary for optimal signal transmission, and ATP is the primary source of this energy. ATP is produced by mitochondria through oxidative phosphorylation of the electron transport chain and glycolysis (Frenzel et al., 2010). Neurons are particularly dependent on mitochondrial ATP as they have reduced levels of 6-phosphofructo-2-kinase/fructose-2, 6-bisphosphatase-3 activity (Pfkfb3), resulting in the shunting of glucose into the pentose-phosphate pathway at the expense of glycolysis (Herrero-Mendez et al., 2009; Bolanos et al., 2010). Levels of ATP were reduced in optic nerves of 6 month old D2 mice in proportion to IOP elevation, and the ability of the compound action potential to recover after oxygen-glucose deprivation was worse in mice with higher IOP levels, suggesting the rate of ATP generation was reduced in these mice to the level where it interfered with transmission of the visual signal along the optic nerve (Baltan et al., 2010). This sensitivity occurred before changes in axon structure (Inman et al., 2006) or anterograde transport were detected (Dengler-Crish et al., 2014). Mitochondrial remodeling was found early in humans with glaucoma (Tribble et al., 2019) and the D2 glaucoma model (Cwerman-Thibault et al., 2017). Rat RGCs showed a sustained decrease in ATP production with IOP elevation that was maintained after IOP levels returned to baseline (Wu et al., 2015). These observations support the theory that mitochondrial dysfunction and ATP reduction are among the first changes that occur following IOP elevation and may be maintained.

In addition to reducing ATP levels, mitochondrial dysfunction also leads to increased generation of reactive oxygen species (ROS), and oxidative stress. Reduced cytochrome c oxidase (Complex IV) activity generates dysfunctional mitochondria, which in turn induces ROS production from the endoplasmic reticulum (ER) (Leadsham et al., 2013; Murphy, 2013). Consequently, the accumulation of dysfunctional mitochondria induces non-physiological ROS production, and the resulting oxidative stress can induce glaucomatous damage (Nita and Grzybowski, 2016). Mitochondrial dysfunction also drives the release of cytochrome c; while cytochrome c is normally involved in the electron transport chain, it can initiate a caspase protease cascade during apoptosis (Chandra et al., 2002; Calkins, 2012). Although apoptosis contributes to RGC degeneration in glaucoma, inhibition of apoptosis is not sufficient to prevent optic neuropathy (Libby et al., 2005). Overall, mitochondrial dysfunction contributes to glaucomatous neurodegeneration by decreasing levels of ATP, increasing ROS generation through reduced Complex IV generation, and defective pathogenesis.

## Hypoxia Contributes to Mitochondrial Dysfunction

IOP elevation exerts a mechanical stretch injury and strain to the tissues of the ONH, pressing the central retinal artery as it passes through the ONH; the subsequent impairment of ocular blood flow reduces the oxygen supply to the retina and induces a localized hypoxia (Josef et al., 2002; Dai et al., 2012; Stowell et al., 2017). As oxidative phosphorylation is dependent on oxygen, prolonged hypoxia results in decreased mitochondrial ATP production. During intermittent hypoxia, the cell can switch from oxidative phosphorylation to glycolysis until oxygen level returns to normal; RGCs exposed to intermittent hypoxia are thus protected from degeneration in ischemic preconditioning (Gidday et al., 2015). In prolonged hypoxia, however, glycolysis is insufficient to meet the high energy demand of neurons. Hypoxia stimulates superoxide generation from Complex III of the electron transport chain. Superoxide is converted to H_2_O_2_ by superoxide dismutase, triggering hypoxia-inducible factor 1α (HIF-1α) stabilization and upregulation (Chandel et al., 1998; Chandel et al., 2000; Hamanaka and Chandel, 2009). Under physiological conditions, hypoxia is resolved by relief of oxidative stress, a metabolic switch to glycolysis, and removal of damaged mitochondria through mitophagy (Wu et al., 2016). However, prolonged hypoxia during glaucoma introduces dysfunctional feedback, impairing mitophagic induction and amplifying the accumulation of dysfunctional mitochondria that result in exacerbated oxidative stress and inflammation. Evidence exists for hypoxia at early stages of glaucoma in the D2 and microbead models (Jassim et al., 2021), and for oxidative stress (Jassim and Inman, 2019), mitochondrial dysfunction and limited mitophagy (Coughlin et al., 2015; Kleesattel et al., 2015) in glaucoma models.

Metabolic vulnerability also contributes to glaucomatous degeneration (Inman and Harun-or-Rashid, 2017; Williams et al., 2017; Harun-or-Rashid et al., 2018; Harun-or-Rashid et al., 2020). Axons rely primarily on glycolysis during glaucoma to compensate for mitochondrial dysfunction, though glycolysis is ultimately insufficient to rescue metabolic vulnerability associated with glaucoma (Jassim et al., 2021). Interestingly, the increased oxidative phosphorylation accompanying a ketogenic diet rescued RGC degeneration due, at least in part, to a reduction in inflammation (Harun-or-Rashid and Inman, 2018).

## Inflammation in Glaucoma

Inflammation is now recognized as a key component of glaucoma neurodegeneration, and increased inflammatory signaling is one of the first changes detected in glaucoma. Activation of localized innate inflammatory signaling is of particular relevance in glaucoma, with involvement of cytokines and complement pathways clearly demonstrated at multiple stages of disease progression (Tezel, 2011; Rieck, 2013; Mac Nair and Nickells, 2015; Kamat et al., 2016; Russo et al., 2016; Bell et al., 2018). The elevated IOP in neovascular glaucoma is associated with high levels of vascular endothelial growth factor (VEGF), and anti-VEGF compounds are used for treatment (Platania et al., 2015; Slabaugh and Salim, 2017). Pro-inflammatory cytokine signaling is also evident in the models; for example, signs of inflammation are present throughout RGC compartments in D2 mice early, change with age, and drive glaucoma in the absence of elevated IOP (Wax et al., 2008; Bosco et al., 2011; Bosco et al., 2015; Wilson et al., 2015). Blocking inflammatory responses has shown promise in ameliorating glaucoma in models (Bosco et al., 2008; Howell et al., 2011; Bosco et al., 2012; Yang et al., 2016; Panchal et al., 2017; Harun-or-Rashid and Inman, 2018), emphasizing the negative impact of inflammation. Induced models of ocular hypertension and optic nerve crush models have also demonstrated inflammation (Morzaev et al., 2015), while inflammation was reported within 4–6 h in the retina after transient IOP elevation (Albalawi et al., 2017; Pronin et al., 2019). RGCs showed mechanosensitive release of multiple cytokines (Lim et al., 2016), while optic nerve head astrocytes showed rapid upregulation and release of IL-6 in response to IOP elevation (Lu et al., 2017). Glaucomatous human eyes and aqueous humor had increased markers for inflammatory cytokines and TNFα (Yang et al., 2011; Takai et al., 2012; Wang et al., 2018).

The NOD-, LRR- and pyrin domain-containing protein 3 (NLRP3) inflammasome is particularly important to inflammatory signaling in glaucoma (Yerramothu et al., 2018). Inflammasomes are multiprotein complexes that can release pro-inflammatory cytokines and are members of Nod-Like Receptor (NLR) or pyrin and HIN domain-containing families (Guo et al., 2015). NLRs are encoded by 23 genes, but only NLRP1, NLRP2, NLRP3, NLRP6, NLRP12, and NLRC4 are capable of forming oligomeric complexes that can activate caspase-1 (CASP1) (Zheng et al., 2020). Inflammasome complexes are composed of cytosolic pattern recognition receptors (PRRs), CASP1, NLRP, and the adaptor protein apoptosis-associated speck-like protein containing a caspase activation and recruitment domain (ASP) (Swanson et al., 2019). The NLRP3 inflammasome is the most widely studied within a glaucoma context, and involvement involves both priming and activation steps. Inflammasome priming occurs through the activation of NFκB signaling (Jo et al., 2016); expression of inflammasome components is low under baseline conditions, and priming to increase expression is necessary for a response. The second step involving assembly and activation of the complex occurs in response to a stressful event; ASC fibrils are recruited and activate CASP1; the accumulation of detectable ASC clusters is a marker for inflammasome activation (Venegas et al., 2017). Activated CASP1 mediates the cleavage of IL-1β and IL-18 into releaseable forms that exit cells through gasdermin D (GSDMD), and in some cases triggering inflammatory cell death through pyroptosis (Liu et al., 2016).

Damage-associated molecular patterns (DAMPs) and pathogen-associated molecular patterns (PAMPs) are common triggers of inflammasome activation (Jo et al., 2016) and DAMPs, such as extracellular ATP and ROS can be released following cell damage (Yin et al., 2016). Extracellular ATP is a widespread mechanism to activate the NLRP3 inflammasome (Couillin et al., 2013), and ATP release is frequently triggered by mechanosensitive changes in tissues, thus providing a potential link between mechanical strain and inflammation (Ventura et al., 2019). This has particular relevance for glaucoma as ATP was elevated in the aqueous humor of humans with acute (Zhang et al., 2007) and chronic angle-closure glaucoma (Li et al., 2011). Increased levels of extracellular ATP accompanied the sustained elevation of IOP in rats following injection of hypertonic saline into episcleral veins, the Tg-MYOCY437H transgenic mouse model, and primates subjected to the laser photocoagulation of the trabecular meshwork (Lu et al., 2015). ATP release was induced from bovine retinal eyecups by elevated pressure (Reigada et al., 2008), and from ONH astrocytes subjected to moderate cyclic strain (Beckel et al., 2014). Under normal conditions, extracellular ATP is rapidly degraded by the ectonucleotidases (Reigada et al., 2005; Allard et al., 2017), but the involvement of ATP in glaucomatous RGC loss suggests that release levels can overwhelm this degradation in some cases (Sanderson et al., 2014).

A role for NLRP3 inflammasome involvement in the loss of RGCs associated with elevated IOP has been demonstrated by multiple groups. Intravitreal injection of ATP triggered significant IL-1β release and ASC speck induction in RGCs and astrocytes, supporting the detrimental effects of extracellular ATP in inflammasome activation (Pronin et al., 2019). Acute activation of NLRP1/NLRP3, CASP1, and IL-1β in mouse RGCs, astrocytes, and Müller glia was detected within 6 h of transient elevation IOP to 120 mmHg, with activation peaking after 12–24 h. Simultaneously, the pyroptotic pore was induced in the ganglion cell layer (GCL) and inner nuclear layer (INL) (Pronin et al., 2019). RGC degeneration was reduced in CASP1/CASP4 knockout (KO) and Panx1 KO mice, and by inhibition of pannexin, suggesting Panx1 activates the inflammasome following ATP release from ischemically or mechanically stressed cells. In a separate study, production of IL-1β following IOP elevation to 110 mmHg for 60 min was attributed to CASP 8 and the NLRP1/NLRP3 inflammasome (Chi et al., 2014). ASC, CASP1, and IL-1β rose in the retina following partial optic nerve crush, while RGC survival was greater when crush was performed in NLRP3 KO mice as compared to control (Puyang et al., 2016). ASC specks were increased in capillaries of contralateral normotensive eyes (Pronin et al., 2019) in addition to the hypertensive eyes; this may relate activated microglia in contralateral normotensive eyes (Rojas et al., 2014).

## Glia Contribute to Inflammatory Responses in Glaucoma

Astrocytes, microglia, and Müller cells are the three major types of retinal glial cells, with the contribution by astrocytes and microglia particularly relevant to inflammation found with glaucoma (Wei et al., 2019; García-Bermúdez et al., 2021). Microglia are innate immune cells residing throughout the retina, ON, and brain. Microglia act as sensors and are one of the first responders following CNS injury, undergoing rapid morphologic and molecular changes as they become “activated” (Lannes et al., 2017). Some forms of activated microglia have beneficial actions, such as increased phagocytosis of toxic debris and release of anti-inflammatory signals (Chen and Trapp, 2016). However, microglia are a key source of inflammatory signals, with prolonged injury leading to excess production of pro-inflammatory cytokines and neurotoxic factors such as IL-6, Tumor necrotic factor-alpha (TNFα), NO, and superoxide (Rodríguez-Gómez et al., 2020). The microglia pro-inflammatory response is coupled with a decrease of the anti-inflammatory cytokine IL-10 during neurodegeneration that aggravates inflammation (Hickman et al., 2008; Heneka et al., 2013).

Reactive microglia have been localized to the retina and ON in multiple glaucoma models, and in human glaucoma (Yuan and Neufeld, 2001; Bosco et al., 2008). Microglial activation is detected in 3 month old D2 mice (Bosco et al., 2011; Bosco et al., 2012), and is predictive of subsequent neurodegeneration (Bosco et al., 2015). Early astrocyte reactivity and microglia activation were shown in the ON of D2 mice, and in rats with OHT following Translimbal Laser Photocoagulation (Son et al., 2010). Early microglial activation, NF-κB signaling, and neuroinflammation in the ONH were also reported in a cat genetic glaucoma model (Oikawa et al., 2020). Minocycline treatment and irradiation inhibited microglial activation and reduced RGC death in D2 mice (Bosco et al., 2008; Bosco et al., 2012), supporting a negative impact of activated microglia on glaucoma progression. Recently, activated microglia were shown to induce reactive neurotoxic astrocytes by the release of interleukin-1 alpha (IL-1α), TNFα, and the classical complement component (C1q), and consequently drive RGC degeneration in the microbead glaucoma model (Liddelow et al., 2017; Guttenplan et al., 2020). Collectively, these studies provide strong evidence of the detrimental impact of activated microglia in glaucoma.

Glia-neuron interaction is emerging as a critical factor in neurodegeneration, and the pivotal role of ATP and purinergic signaling links cellular energetics to this interaction. Microglia constantly regulate and influence neurons via specialized somatic junctions (Madry et al., 2018; Cserep et al., 2020). ATP leakage from injured cells, through mechanosensitive channels, or from neuronal mitochondria through vesicular nucleotide transporter (vNUT) channels enriched at microglia-neuron contact sites is sensed by P2Y12 receptors on microglia, triggering process extension and migration toward the injured sites (Koizumi et al., 2013; Cserep et al., 2020). Whether P2Y12 receptors play a direct role in microglial surveillance, or potentiate the activity of THIK-1 potassium channels as recently suggested (Madry et al., 2018), P2Y12 receptor stimulation by ATP clearly contributes to surveillance. Stimulation of the P2X7 receptor has also been implicated in microglial phagocytosis and degradation (Campagno and Mitchell, 2021), an effect which may have particular impact in aging cells. Inhibition of the P2X7 receptor was shown to reduce microglia activation in D2 mice (Romano et al., 2020), suggesting a key role for the receptor in the inflammatory response in glaucoma. The P2X7 receptor also induces a rise in ROS (Bartlett et al., 2013; Munoz et al., 2017); whether this provides a pathway to link mitochondria with inflammation in glaucoma remains to be determined.

Optic nerve head astrocytes are also implicated in the link between mechanical strain and inflammation. Stretch and swelling of ONH astrocytes led to the release of ATP through pannexin hemichannels (Beckel et al., 2014). Stimulation of this released ATP through pannexins was implicated in the priming of the NLRP3 inflammasome, with increased expression of IL-1β, NLRP3 and caspase1 (Albalawi et al., 2017). Transient elevation of IOP led to a similar priming and release of IL-6 from optic nerve head astrocytes as well as ganglion cells (Lu et al., 2017).

Signaling from neurons back to glia also contributes to the link between mitochondrial dysfunction and inflammation in glaucoma. For example, fragmented and damaged mitochondria are found in activated microglia as a result of increased mitochondrial fission (Joshi et al., 2019). These damaged mitochondria are released into extracellular space, inducing an innate immune response by targeting adjacent astrocytes can also release dysfunctional mitochondria (Joshi et al., 2019). The resulting positive feedback can accelerate neuroinflammation. Inhibiting mitochondrial fission with heptapeptide P110, which inhibits binding of Drp1 to the mitochondrial receptor Fis1, reduced fragmentation and mitochondrial release from microglia, lessened astrocyte activation, and protected neurons from innate immune attack. Extracellular mitochondria can also signal between glia and neurons; functional mitochondria were found to be protective, while damaged mitochondria communicated pathology following stroke (Hayakawa et al., 2016). This suggests that the health of released mitochondrial may influence pathology in glaucoma.

Astrocytes are generally considered to protect neurons from oxidative stress, specifically via glutathione precursor synthesis, as they have strong antioxidant defenses regulated by the transcription factor Nrf2, a master regulator of redox homeostasis (Shih et al., 2003; Himori et al., 2013; Ghosh et al., 2020). However, reactive astrocytes contribute to neuronal degeneration in mice with sustained IOP elevation and reduction of their activated status rescued neuronal function (Guttenplan et al., 2020; Sterling et al., 2020). The decline in astrocytic antioxidant defense mechanisms and the increase in astrocytic reactivity during glaucoma occur simultaneously with mitochondria dysfunction, contributing to ROS accumulation and oxidative stress that enhance glaucoma progression (Tezel, 2006; Jassim and Inman, 2019). Intravitreal injection of neurotoxic astrocytes did not induce RGC neurodegeneration in the absence of neuronal injury, suggesting that injury and glial activation are required for neurodegeneration (Guttenplan et al., 2020).

## Crosstalk Between Mitochondrial Dysfunction and Inflammation

Glaucoma is a complicated and progressive neurodegenerative disease where multiple pathways contribute to pathogenesis. Given that mitochondrial dysfunction and inflammation are two of the most potent influences, emerging evidence for interactions between these two factors has relevance for the etiology of glaucoma.

## Mitochondrial Dysfunction Contributes to Inflammation

Mitochondrial dysfunction and inflammation are interdependent processes. Inhibition of Complex I by rotenone, or of Complex III by antimycin A, in bone marrow-derived macrophages and in primary mouse microglia (Ferger et al., 2010) induced oxidative stress, activated microglia, activated the NLRP3 inflammasome, and increased IL-1β production, resulting in pyroptosis (Zhou et al., 2011). Rotenone administration concomitant with inhibition of autophagy caused the accumulation of damaged mitochondria with downstream IL-1β production (Nakahira et al., 2011; Zhou et al., 2011). Furthermore, subcutaneous injections of rotenone in rats increased IL-1β within the hypothalamus, confirming that mitochondria may act upstream of inflammation (Yi et al., 2007). Antioxidant treatment using sulforaphane (SFN) significantly prevented RGC death and suppressed microglia and inflammasome activation in the transient IOP (110 mmHg for 1 h) model in rats suggesting that ROS production is upstream of inflammation (Gong et al., 2019). Collectively, studies indicate that mitochondria play an important role in regulating inflammation and that mitochondrial dysfunction is upstream of inflammation (Misawa et al., 2013); this has yet to be determined in glaucoma, however.

Mitochondrial dysfunction may contribute to various forms of inflammatory signaling, with links to NLRP3 inflammasome signaling of particular relevance for neurodegeneration (Nakahira et al., 2011; Zhou et al., 2011; Gurung et al., 2015). Many of these pathological links are related to excess levels of ROS; while ROS are mainly generated as byproducts of oxidative phosphorylation, excess production or inadequate removal of ROS can result in oxidative stress. Accumulated ROS results in the opening of the mitochondrial permeability transition pores that facilitates release of ROS (Kozlov et al., 2017) and damaged mtDNA (Shimada et al., 2012) into the cytoplasm; both substances act as DAMPs to induce NLRP3 inflammasome activation and pyroptosis (Latz et al., 2013; Yin et al., 2016; Bai et al., 2018). As ROS are short-lived and act only across short distances (Veal et al., 2007), positional shifts that recruit NLRP3 towards mitochondria enhance the ability of ROS to increase NLRP3 activation. During activation of the NLRP3 inflammasome, NLRP3 redistributes from the ER to mitochondria-associated ER membranes (MAMSs), where NLRP3 connects to the ASC adaptor protein, localized on the mitochondria, enabling inflammasome assembly (Green et al., 2011; Misawa et al., 2013; Heid et al., 2013; Misawa et al., 2013). Although the approximation of NLRP3-ASC at MAMs is important for NLRP3 activation, other factors also contribute. For example, the mitochondria-associated adaptor molecule, MAVS, is required for NLRP3 inflammasome activity as it promotes the recruitment of NLRP3 to the mitochondria and the subsequent IL-1β production *in vivo* (Subramanian et al., 2013), however, this has yet to be shown in glaucoma.

Mitochondrial dysfunction can also lead to increased inflammatory signaling through the cyclic GMP–AMP synthase (cGAS)–stimulator of interferon genes (STING) pathway (West and Shadel, 2017). The enzyme cGAS detects cytoplasmic DNA, including mtDNA leaked from damaged mitochondria. The reaction product cGAMP activates STING (Gao et al., 2013), which in turn stimulates TANK-binding kinase 1 (TBK1), to promote homodimerization of interferon regulatory factor 3 (IRF3) (Tanaka and Chen, 2012). Nuclear translocation of this phosphorylated IRF3 enhances expression of interferons and an enlarged interferon response (Hopfner and Hornung, 2020). mtDNA released across the plasma membrane can activate cGAS- or TLR9-dependent interferon signaling, thus communicating the mitochondrial damage to neighboring cells (West and Shadel, 2017). Components of the cGAS-STING pathway have been identified in the murine retina (Tang et al., 2019). In retinal microvascular endothelial cells, mtDNA in the cytosol stimulated the cGAS-STING pathway and nuclear translocation of IRF3 (Guo et al., 2020). Mutations in optineurin (OPTN) associated with primary open angle glaucoma (E50K) reduced the phosphorylation of IRF3 and IFNα/β release assays in response to poly (I:C) stimulation of TLR3 (O'loughlin et al., 2020). Further investigations into the interactions between mtDNA releases as a result of mitochondrial dysfunction in glaucoma and the cGAS-STING-pathway promise to be informative.

Patients with glaucoma have an increased risk of developing Alzheimer’s disease (Moon et al., 2018), and deposits of Alzheimer’s disease marker amyloid beta (Aβ) accumulate in RGCs following IOP elevation (Guo et al., 2007), suggesting interactions between Aβ and mitochondria may contribute to the pathology. Aβ accumulation in mitochondrial cristae negatively impacted mitochondrial function. The translocase of the outer membrane (TOM) machinery moves Aβ across the membrane, allowing it to accumulate (Petersen et al., 2008). Human neuroblastoma cells also internalized extracellularly applied Aβ that colocalized with mitochondrial markers (Petersen et al., 2008). While Aβ accumulation has been shown in several glaucoma models (Mckinnon et al., 2002; Guo et al., 2007; Wilson et al., 2016), the accumulation of Aβ in neuronal and glial mitochondria has yet to be shown in glaucoma as it has in the brain.

Mitochondrial dysfunction drives metabolic vulnerability in the D2 mouse ON and retina, which in turn triggers AMP-activated protein kinase activation (AMPK), a cellular energy sensor, to activate NF-κB signaling and increase expression of inflammatory genes (Harun-or-Rashid and Inman, 2018). Treatment with a ketogenic diet reduces inflammation, possibly while inhibiting AMPK activation while also meeting the high neuronal energy demand. Expression of AMPK was upregulated in the RGC of mice with elevated IOP following injection of magnetic microbeads (Belforte et al., 2018). Additional exploration of the role of AMPK in connecting mitochondrial damage with inflammation in glaucoma is likely to be fruitful, given the role of AMPK in systemic disease, and the therapeutic potential of manipulating this pathway in ocular disease (Powell et al., 2020).

## Hypoxia Contributes to Inflammation

The NLRP3 inflammasome can also link hypoxia to inflammation and suggests how hypoxia, and thus increased IOP in glaucoma, can contribute to inflammation. Chronic intermittent hypoxia increased levels of cytokines associated with M1-and M2-like microglial activation states (Snyder et al., 2017). In retinal pigmented epithelial cells, hypoxia induced expression of NLRP3 and IL-1β in a pathway dependent upon ATP release and the P2Y12 receptor, and inflammasome activation killed cells only under hypoxic conditions (Doktor et al., 2018). HIF-1α is implicated in hypoxia-mediated inflammasome priming as blockage of HIF-1α reduced expression of NLRP3, caspase 1 and IL-1β and of pyroptotic death in a stroke model (Jiang et al., 2020).

Similar connections between hypoxia and inflammation may occur in glaucoma. IOP elevation and hypoxia can induce pyroptosis by activating CASP8; CASP8 triggered NF-kB translocation to induce HIF-1α signaling, which in turn facilitated NLRP12/NLRP3/NLRC4 assembly and activation *in vitro* and *in vivo* (Chen et al., 2020). Hypoxia also induces CASP1 release, NLRP3 inflammasome activation, IL-1β release, GSDMD cleavage, and pyroptosis (Watanabe et al., 2020). NLRP3 deficiency and CASP1 blockade significantly inhibited hypoxia-induced IL-1β release from macrophages. Indeed, genetic deletion of GSDMD, CASP8, or NLRP12 reduced RGC death after the transient IOP elevation model, where IOP was elevated to 110 mmHg for 90 min (Chen et al., 2020).

Given that oxidative phosphorylation in the mitochondria is dependent on oxygen and is the main source of cellular ATP in addition to glycolysis, hypoxia and glucose deprivation decrease ATP, facilitate K^+^ efflux, and induce IL-1β release. Interestingly, these effects were reversible by K^+^ efflux inhibition and K_ATP_ channel blockers in macrophages (Watanabe et al., 2020). The NLRP3 inflammasome acts as an intercellular sensor of ATP decrease induced by glucose and oxygen deprivation (Watanabe et al., 2020). Of relevance were studies showing that K_ATP_ channel opener KR-31378 protected RGCs from ischemic damage (Bucolo et al., 2018). In retinal vessels activation of the K_ATP_ channel dramatically increased the vasotoxicity of P2X7 receptor stimulation through elevation of calcium and increased oxidative stress (Shibata et al., 2018); such interactions may increase hypoxic challenge in glaucoma given the propensity of excess P2X7 receptor stimulation with elevated IOP (Mitchell et al., 2008). Further studies will be necessary to elucidate the precise link between the K_ATP_ channel, the P2X7 receptor, NLRP3 inflammasome activation and cellular metabolic crisis in glia vs. neurons during glaucoma.

There is also considerable evidence of a role for carbon monoxide in glaucoma (Bucolo and Drago, 2011). A carbon monoxide-releasing molecule, CORM-3, produced a dose-dependent reduction in IOP in the rabbit eye (Stagni et al., 2009). The precise mechanism remains to be determined, although action on KCa^2+^ channels in the outflow pathway has been suggested (Dong et al., 2007; Bucolo and Drago, 2011). Recent work in a model of hind limb ischemia suggests the stabilization of HIF-1α by hemeoxygenase 1 (Hmox1) is at least partially attributed to carbon monoxide, with carbon monoxide a by-product of the breakdown of heme by of Hmox1 (Dunn et al., 2021). In addition, carbon monoxide regulates mitochondrial biogenesis and gene expression, suggesting multiple protective sites in glaucoma are possible (Cherry and Piantadosi, 2015).

## Dysfunctional Mitophagy Exacerbates Inflammation

Mitophagy helps regulate mitochondria homeostasis by getting rid of dysfunctional mitochondria, and the inhibition of mitophagy results in the accumulation of damaged mitochondria and sometimes inflammasome activation. Mitophagy is driven by PTEN-induced putative kinase 1 (PINK1) and parkin (E3 ubiquitin ligase), where the cytoplasmic Parkin is recruited to the mitochondria to interact with PINK1 on the outer mitochondrial membrane and target dysfunctional mitochondria (Palikaras et al., 2018). Adaptor proteins such as p62 and OPTN join poly-ubiquitinated strands to light chain 3 (LC3), initiating autophagy. The mitochondrial accumulation of LC3 puncta after treatment with Complex I inhibitor rotenone indicate mitophagy is increased by mitochondrial stress (Zhou et al., 2011).

Impaired mitophagy has been implicated in glaucoma by multiple observations. Elevation of IOP in rats increased damaged mitochondria, parkin and optineurin levels in RGCs, while function was partially restored following overexpression of Parkin (Dai et al., 2018). Impaired mitophagy was also implicated in the myelinated ON axons of D2 mice by a rise in fragmented and damaged mitochondria without changes in PINK or parkin levels (Coughlin et al., 2015). These mice also displayed increased mitochondria within autophagosomes in distal and proximal axons (Kleesattel et al., 2015). An autosomal dominant form of normal tension glaucoma is linked to mutations in OPTN (Rezaie et al., 2002), and mice transgenic for E50K, the most common mutation in normal tension glaucoma, showed altered mitophagy and mitochondrial fission (Shim et al., 2016). Pink1 and Parkin KO mice both showed an increase in increased inflammation, but antioxidants abolished CASP1 activation, suggesting a role for ROS in the inflammation associated with impaired mitophagy (Sliter et al., 2018). These findings emphasize the importance of mitophagy in combating inflammation, and justify further examination in glaucoma.

NLRP3 inflammasome activation is negatively regulated by mitophagy (Latz et al., 2013; Lai et al., 2018). Autophagic proteins contribute to an anti-inflammatory response by regulating NLRP3 inflammation and mitochondrial integrity (Nakahira et al., 2011). Inflammasome activation recruits autophagy adaptor protein p62 to the mitochondria. Measuring LC3 and p62 puncta is a method of quantifying autophagy/mitophagy. LC3 and p62 enable mitophagy, thereby inhibiting NLRP3 inflammasome activation and preventing excessive IL-1β production by degrading damaged mitochondria in macrophages (Zhong et al., 2016). Depletion of genes for autophagic proteins (specifically LC3B and Beclin 1), and the use of mitophagy inhibitors (such as 3-methyladenine), promoted CASP1 activation, secretion of IL-1β and IL-18, and the accumulation of dysfunctional mitochondria in macrophages and *in vivo* (Nakahira et al., 2011). In addition, stimulation by lipopolysaccharide (LPS) or ATP led to the release of mtDNA and ROS into the cytosol and inflammasome-dependent secretion of IL-1β and IL-18 (Nakahira et al., 2011).

Mitophagy can limit apoptosis by reducing the accumulation of dysfunctional mitochondrial and oxidative stress, and facilitate the metabolic switch of the cell from oxidative phosphorylation to glycolysis to adapt to the hypoxia reported during glaucoma (Liu et al., 2012a; Jassim and Inman, 2019). Hypoxia-induced mitophagy occurs through the action of a mitochondrial associated membrane protein, FUNDC1, as reported *in vitro* (Liu et al., 2012a; Chen et al., 2016; Wu et al., 2016). During hypoxia, oxidative phosphorylation is expected to decline and glycolysis would become the primary ATP source in the cell. Although high reliance on glycolysis was recently shown in glaucomatous D2 ON (Jassim et al., 2021), degeneration proceeds, indicating that ATP produced from glycolysis is insufficient to meet the high energy demand of axons during glaucoma.

## Inflammation can Induce Mitochondrial Dysfunction

While there is considerable evidence suggesting that dysfunctional mitochondria can trigger inflammation, the opposite is also true, with inflammation inducing mitochondrial dysfunction. Inflammasome assembly can impair organelle function and integrity; for example, activation of the NLRP3 inflammasome in macrophages reduced cytoplasmic levels of ATP and mitochondrial function (Heid et al., 2013). Inflammation in LPS-treated macrophages resulted in a metabolic shift from oxidative phosphorylation to glycolysis (Mills et al., 2016). Interestingly, Complex II and Complex I oxidation, and decreased NAD^+^ were necessary for the pro-inflammatory response observed in these macrophages. TNFα induced a oxidative phosphorylation deficit in a mouse hippocampal cell line, suggesting a detrimental impact of inflammation on mitochondrial function (Doll et al., 2015). In optic nerve head astrocytes, stimulation of TLR3 led to a transfer of cellular ATP from cytoplasmic to extracellular compartments, suggesting inflammatory signaling can strain cellular energetics in relationship to glaucoma (Beckel et al., 2018).

Microglia metabolic reprogramming has been found in response to inflammation as cells switch between oxidative phosphorylation and glycolytic metabolism (Lauro and Limatola, 2020). Activated microglia have dysfunctional mitochondria and they switch to glycolysis to compensate for ATP loss. Stimulation of microglial cells with LPS reduced mitochondrial oxygen consumption, ATP production and oxidative phosphorylation, while increasing glycolysis (Voloboueva et al., 2013). In addition, mitochondrial dysfunction in microglia propagates mitochondrial dysfunction in neurons and can block some of the alternative response triggered by IL-4 (Ferger et al., 2010). As this IL-4 response can reduce inflammation, mitochondrial dysfunction might contribute to the pathological changes found in activated microglia in glaucoma. Whether inflammatory stimuli lead microglia in the retina to switch from oxidative phosphorylation to glycolysis should be investigated given central role of microglia in glaucomatous pathogenesis.

## Discussion and Future Directions

**FIGURE 1:**
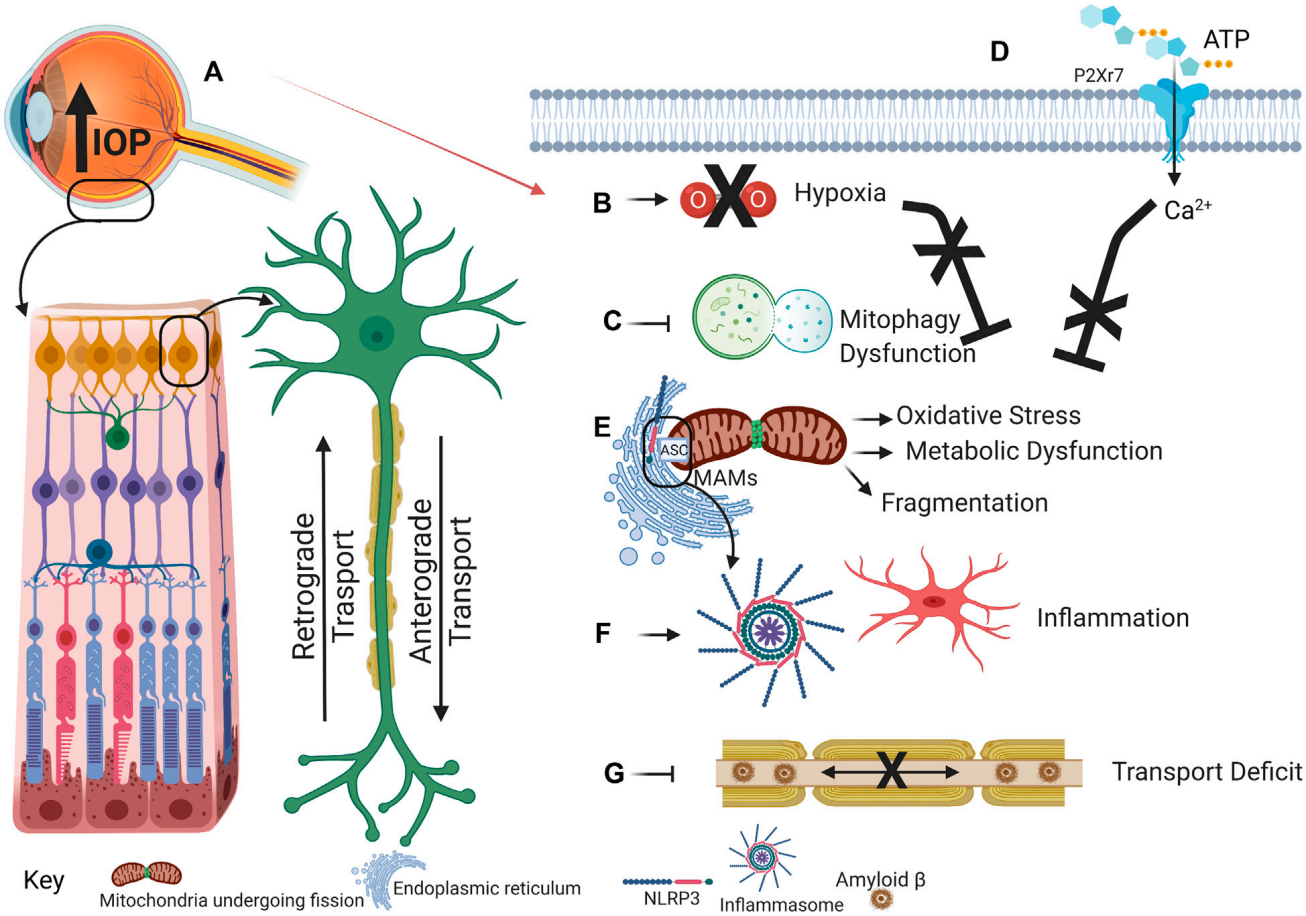
This schematic illustrates the proposed cascade of events that link the increased intraocular pressure of glaucoma to mitochondrial dysfunction and the NLRP3 inflammasome. **(A)** Intraocular pressure increases as a result of perturbed aqueous humor outflow in the eye. **(B)** Intraocular pressure increase prevents efficient blood flow to the eye, resulting in hypoxia/ischemia. During physiological conditions, hypoxia induces mitophagy to eliminate dysfunctional mitochondrial accumulation. **(C)** However, during glaucoma, hypoxia-induced mitophagy may be inhibited. Concomitantly, external ATP binds to and opens the cation-selective P2X7R **(D)**. Ca^2+^ influx can stimulate mitochondrial dysfunction that will induce inflammation, activated microglia, and cytokine release. **(E)** Dysfunctional and fragmented mitochondria accumulate, thus promoting oxidative stress and metabolic vulnerability. Oxidative stress induces inflammasome assembly and activation as NLRP3, localized at the ER, comes into proximity to the ASC, localized at the mitochondria, on MAMs. **(F)** Inflammasome activation releases cytokines that establish a positive feedback loop to exacerbate further inflammatory response. **(G)** Aβ that has been demonstrated in mitochondria can also accumulate in axons, thereby inducing axon transport deficit that further contributes to glaucoma progression. Created with Biorender.com

The strong support for mitochondrial dysfunction and inflammation in glaucoma outlined above, combined with growing evidence for crosstalk between mitochondrial dysfunction and inflammation in other neurodegenerations, suggests interaction between these processes contributes to the expanding pathogenesis in glaucoma patients. We propose that IOP elevation initiates hypoxia that contributes to mitochondrial dysfunction, oxidative stress, impaired mitophagy and inflammation and that these processes are exacerbated by interactions between inflammation and mitochondrial dysfunctional (Figure 1).

While expanding evidence for both inflammation and mitochondrial dysfunction supports crosstalk, the degree of interaction may be influenced by several key factors, and suggests several key targets for intervention (Table 1). For example, the microglial activation state is expected to have a considerable impact on waste accumulation and impaired mitophagy (Campagno and Mitchell, 2021). Investigations into compartmentalized interaction between ASC, NLRP3 and oxidative stress in soma, axon, and synapse has particular relevance for glaucoma given the ganglion cell architecture. The ability of inflammation to disrupt mitochondrial signaling remains largely undetermined in glaucoma. The development of *in vitro* models using neurons, astrocytes, and microglia, in addition to the use and development of mouse glaucoma models with knockout technologies, will enable us to resolve these questions.

**TABLE 1:** Pharmacological targets to ameliorate mitochondrial dysfunction and inflammation.

Protein/Gene	Biological target	Targeted pathway	Scientific evidence	References
HIF-1α	Nucleus	Hypoxic response	Hypoxia preconditioniong rescue RGC during glaucoma	Gidday et al. (2015)
K_ATP_ channel	Membrane	Metabolic function	K_ATP_ blockers reduced IL-1β release; K_ATP_ opener protected RGCs from ischemic damage	Watanabe et al. (2020)
HCAR1	Mitochondria	l-lactate receptor	Ketogenic diet stimulates HCAR1 to inhibit NLRP3 inflammasome in glaucoma	Harun-or-Rashid and Inman, (2018)
AMPK	Cytosol	Energy sensor protein kinase	Ketogenic diet reduced metabolic vulnerability and AMPK-induceds inflammation	Harun-or-Rashid and Inman, (2018)
cGAS	Interferon in cytosol	STING pathway	Detects leaked mtDNA	Sintim et al. (2019)
Aβ	Cytosol, mitochondria	Biomarker of neurodegeneration, impaired clearance	Accumulates in mitochondria cristae, blocks function; induces pro-inflamatory cytokines via P2X7R	Chiozzi et al., 2019, Petersen et al., 2008
